# Recent developments in immunotherapy of acute myeloid leukemia

**DOI:** 10.1186/s13045-017-0505-0

**Published:** 2017-07-25

**Authors:** Felix S. Lichtenegger, Christina Krupka, Sascha Haubner, Thomas Köhnke, Marion Subklewe

**Affiliations:** 1 Department of Medicine III, University Hospital, LMU Munich, Germany; 2 Laboratory of Translational Cancer Immunology, Gene Center, Munich, Germany; 3 German Cancer Consortium (DKTK), Partner Site, Munich, Germany; 4 German Cancer Research Center (DKFZ), Heidelberg, Germany

**Keywords:** AML, Antibody therapy, Bispecific antibody, CAR T cell, Checkpoint inhibition, Dendritic cell vaccination, Epigenetic therapy, Immunotherapy

## Abstract

The advent of new immunotherapeutic agents in clinical practice has revolutionized cancer treatment in the past decade, both in oncology and hematology. The transfer of the immunotherapeutic concepts to the treatment of acute myeloid leukemia (AML) is hampered by various characteristics of the disease, including non-leukemia-restricted target antigen expression profile, low endogenous immune responses, and intrinsic resistance mechanisms of the leukemic blasts against immune responses. However, considerable progress has been made in this field in the past few years.

Within this manuscript, we review the recent developments and the current status of the five currently most prominent immunotherapeutic concepts: (1) antibody-drug conjugates, (2) T cell-recruiting antibody constructs, (3) chimeric antigen receptor (CAR) T cells, (4) checkpoint inhibitors, and (5) dendritic cell vaccination. We focus on the clinical data that has been published so far, both for newly diagnosed and refractory/relapsed AML, but omitting immunotherapeutic concepts in conjunction with hematopoietic stem cell transplantation. Besides, we have included important clinical trials that are currently running or have recently been completed but are still lacking full publication of their results.

While each of the concepts has its particular merits and inherent problems, the field of immunotherapy of AML seems to have taken some significant steps forward. Results of currently running trials will reveal the direction of further development including approaches combining two or more of these concepts.

## Background

Advances in immunotherapy have revolutionized cancer therapy in the past few years. Novel immunotherapeutic approaches are entering the mainstream of oncology. In hematology, progress has primarily been made in the field of B-lymphoproliferative diseases including acute lymphoblastic leukemia (ALL). In acute myeloid leukemia (AML), novel strategies utilizing the immune system to eliminate leukemic cells have only recently approached clinical application [1, 2]. This is somewhat surprising, considering that allogeneic hematopoietic stem cell transplantation (HSCT) is one of the oldest immunotherapeutic strategies for postremission therapy in AML. So far, HSCT remains the most successful therapy for prevention of relapse in non-favorable risk patients with AML [3, 4]. However, relapse after allogeneic HSCT does occur, and the vast majority of elderly patients are not eligible for HSCT. Therefore, alternative immunotherapeutic strategies are urgently needed to treat patients not suitable for intensive treatment regimens as well as patients with relapsed or refractory (r/r) disease [5].

In ALL, several antibody-based approaches have already entered standard treatment or are at the verge of approval. Rituximab, an anti-CD20 directed antibody has been shown to be beneficial as an additive to conventional chemotherapeutic agents [6]. Inotuzumab ozogamicin is a toxin-conjugated monoclonal antibody directed against CD22 on the surface of B cells. Approval in r/r ALL is expected in the next year after a phase III trial demonstrated 80.7% overall response rate (ORR) [7]. Moreover, novel T cell-recruiting therapies have opened up an entirely new approach to the treatment of acute leukemias, bypassing typical tumor resistance mechanisms [8]. Blinatumomab, a bispecific molecule connecting CD3 in the T cell receptor complex with CD19 expressed by B cells, was the first T cell-recruiting antibody approved for the treatment of cancer in 2014 [9]. Chimeric antigen receptor (CAR) T cells advance this concept even further by engineering a T cell with the specificity of a monoclonal antibody and a T cell activation domain. The engineered T cells are thus capable of targeting surface molecules of tumor cells in their native conformation independently of MHC [10]. In principle, all of these treatment modalities can be translated to AML.

However, targeted immunotherapy relies on a suitable target antigen to avoid unwanted on-target off-tumor toxicity. In ALL, the restricted expression profile of CD19 and CD20 allows to target these B cell-associated antigens. In AML, it is more difficult to choose an appropriate target antigen due to a more ubiquitous expression pattern overlapping with healthy hematopoiesis. Various potential target antigens are studied for each of the immunotherapeutic strategies [11, 12]. Still, it is to be expected that targeting AML-associated antigens will result in prolonged drug-induced cytopenias. This will require the adjustment of current protocols applied in ALL to the different setting in AML.

Other immunotherapeutic concepts rely on the enhancement of endogenous or the priming of new immune responses. Checkpoint inhibitors have been successfully approved in several solid organ malignancies and are now entering the treatment of hematological diseases [13]. And therapeutic vaccines, particularly those based on dendritic cells (DCs), have been shown to reliably induce anti-leukemic immune responses. Combining these two strategies not only with each other but also with hypomethylating agents (HMAs), which have been shown to modulate the immune function, seems suitable.

In this review, we will present recent advances made in the aforementioned fields of immunotherapy of AML. HSCT and immunotherapeutic strategies for relapse after HSCT constitute a review topic on their own and have been excluded. As published data from clinical trials is still scarce for the majority of immunotherapeutic approaches, we will integrate currently running clinical trials to point out upcoming directions in this field.

## Antibody-drug conjugates for immunotherapy of AML

**Fig. 1:**
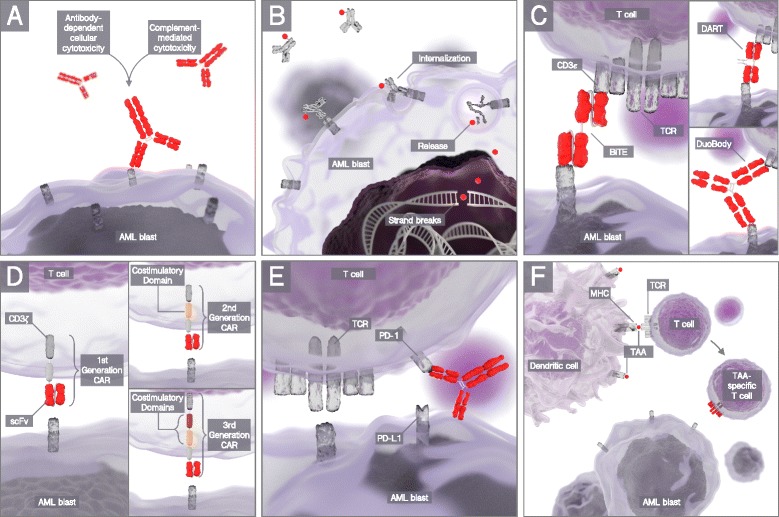
Mechanisms of cancer immunotherapy. Different immunotherapeutic concepts are discussed in the context of AML in this review. **a** Conventional antibodies directed at AML surface antigens mediate antibody-dependent cellular cytotoxicity as well as complement-mediated cytotoxicity. **b** Antibody-drug conjugates consist of monoclonal antibodies conjugated to various toxins, which are released upon internalization and induce cell death through mechanisms like DNA double-strand break and cell cycle arrest. **c** T cell-recruiting antibody constructs are composed of single-chain variable fragments of two antibodies of different specificity connected by a short peptide linker. Their purpose is to bring malignant cells and T cells in close proximity through simultaneous binding of a tumor-associated antigen and CD3ε in the T cell receptor complex. **d** Chimeric antigen receptors (CARs) are genetically engineered cell membrane-bound receptors combining extracellular antibody binding and intracellular effector cell signaling. Their structure enables both MHC-independent antigen binding and highly potent cytotoxic effector cell function. Compared to the first generation of CARs, the introduction of various costimulatory domains in later-generation CAR constructs greatly improved their anti-tumor effector function. **e** Checkpoint inhibitors are monoclonal antibodies binding to inhibitory receptors on T cells or their ligands on antigen-presenting cells or cancer cells, thus boosting the effects of pre-existing T cell responses. **f** Dendritic cells are professional antigen-presenting cells. Vaccination strategies using in vitro*-*generated dendritic cells have the purpose to prime new or enhance pre-existing antigen-specific immune responses

Compared to conventional antibody formats (Fig. 1a), antibody-drug conjugates (ADCs), consisting of monoclonal antibodies conjugated to various toxins, are a tool to bridge conventional chemotherapy and innovative immunotherapy. Upon internalization, the toxin is released in the acidic environment of the lysosomes and reaches the nucleus where it induces cell death through mechanisms like DNA double strand break and cell cycle arrest (Fig. 1b). The prerequisite for successful immunochemotherapy is a rapidly internalizing target antigen, preferably specific to the tumor [14].

CD33 (SIGLEC-3) is the antigen that has been most commonly targeted so far in AML. The first and most prominent ADC in clinical application was gemtuzumab ozogamicin (GO, Mylotarg, Pfizer), a humanized anti-CD33 IgG4 antibody conjugated to calicheamicin. Promising clinical results lead to an accelerated approval of the antibody by the Food and Drug Administration (FDA) in 2000 [15]. Safety concerns and failure to verify clinical benefit in a confirmatory phase III trial enrolling patients across all cytogenetic risk groups resulted in the voluntary withdrawal of GO from the market in 2010 [16]. In recent years, both retrospective analyses and new clinical trials have been performed to unravel clinical benefits of GO in specific subgroups. A meta-analysis of five randomized controlled trials (RCTs) showed that the addition of GO to conventional chemotherapy significantly reduced the risk of relapse and resulted in an overall survival (OS) benefit mainly for cytogenetically favorable as well as for the intermediate-risk group [17]. Another meta-analysis of 11 RCTs with one arm including GO showed improval in OS only for patients with favorable genetics [18]. A recent clinical trial testing GO vs. best supportive care including hydroxyurea in older patients with newly diagnosed AML unsuitable for intensive chemotherapy confirmed the clinical benefit, particularly in those patients with favorable or intermediate cytogenetic risk profile [19].

In order to further improve the clinical results with GO, several clinical trials have been performed evaluating GO in combination with HMAs. A regimen consisting of hydroxyurea, azacitidine, and GO was tested in a phase II trial for 142 older patients with newly diagnosed AML. The predefined goals concerning efficacy and safety were met for the poor-risk cohort (age ≥70 years and performance status 2 or 3), but not for the good-risk group [20]. GO in combination with both the histone deacetylase inhibitor vorinostat and the DNA methyltransferase I inhibitor azacitidine was studied in a phase I/II trial for older patients with r/r AML. An ORR of 41.9% was seen among the 43 patients that were treated at the maximum tolerated dose, which can be considered rather high in this difficult-to-treat cohort [21]. And finally, 110 patients with newly diagnosed or r/r AML or high-risk myelodysplastic syndrome (MDS) were treated with decitabine and GO within a phase II study. Compared to historical controls, ORR was increased, but not OS [22]. Another combination trial with GO and azacitidine for patients with relapsed AML has not yet been reported (NCT00766116, Table 1).

**Table 1:** Current clinical trials using antibody-drug conjugates for immunotherapy of AML

Study identifier	Study name	Antigen/target	Drug name	Combination therapy	Clinical phase	Indication (AML only)	Primary endpoints	(Estimated) Enrollment	Sponsor	Country	Study start	(Estimated) Completion date	Status
NCT00766116	A phase I/II trial of the combination 5-azacitidine and gemtuzumab ozogamicin therapy for treatment of relapsed AML	CD33	Gemtuzumab ozogamicin	Azacitidine	I/II	Relapsed AML	Phase I: MTD; phase II: clinical response (CR rate)	50	University of California, San Diego	USA	2005	2017	Active, not recruiting
NCT01902329	A phase 1 trial of SGN-CD33A in patients with CD33-positive acute myeloid leukemia	CD33	SGN-CD33A	Azacitidine or decitabine	I	Relapsed AML or newly diagnosed AML if not a candidate for intensive chemotherapy; CD33 expression	Toxicity	195	Seattle Genetics	USA	2013	2017	Active, not recruiting
NCT02326584	A phase 1b dose-escalation study of SGN-CD33A in combination with standard-of-care for patients with newly diagnosed acute myeloid	CD33	SGN-CD33A	Standard of care	I	Newly diagnosed AML	Toxicity	144	Seattle Genetics	USA	2014	2017	Active, not recruiting
NCT02674763	A phase 1, multi-center, open-label study of IMGN779 administered intravenously in adult patients with relapsed/refractory CD33-positive	CD33	IMGN779	n.a.	I	r/r AML; CD33 expression	MTD	124	ImmunoGen	USA	2016	2019	Recruiting
NCT02785900	Vadastuximab talirine (SGN-CD33A; 33A) combined with azacitidine or decitabine in older patients with newly diagnosed acute myeloid leukemia (CASCADE)	CD33	SGN-CD33A	azacitidine or decitabine	III	Newly diagnosed AML with non-favorable risk type; not a candidate for allogeneic HSCT	Clinical response (OS)	500	Seattle Genetics	USA, Australia, Korea, Taiwan, various European countries	2016	2021	Recruiting
NCT02848248	A phase 1 study of SGN-CD123A in patients with relapsed or refractory acute myeloid leukemia (AML)	CD123	SGN-CD123A	n.a.	I	r/r AML; CD123 expression	Toxicity	102	Seattle Genetics	USA	2016	2019	Recruiting

As CD33 is expressed on >30% of healthy bone marrow cells, on-target off-leukemia toxicity is inevitable [23–25]. However, a major part of the side effects observed in the clinical trials with GO were attributed to linker instabilities and subsequent off-target toxicities [26, 27]. A lot of effort has therefore been put into the optimization of the ADC technology. An alternative ADC directed against CD33, SGN-CD33A (vadastuximab talirine), has recently entered clinical trials. In this construct, a monoclonal anti-CD33 antibody is conjugated to a highly potent DNA-binding pyrrolobenzodiazepine dimer. The linker technology has been optimized and allows uniform drug loading [28]. Based on promising preclinical data, several clinical trials have been initiated evaluating safety and efficacy of SGN-CD33A alone or in various combinations. Twenty-seven treatment-naive AML patients ineligible for intensive chemotherapy were treated with the recommended monotherapy dose of 40 μg/kg within a phase I study (NCT01902329). The adverse events (AEs) observed were reported to be generally manageable, with a preponderance of myelosuppression. Combined complete remission (CR) and complete remission with incomplete recovery (CRi) rate was 54% [29]. Within another cohort of the same study, 53 patients were treated with a combination of SGN-CD33A and HMAs, resulting in an encouraging CR/CRi rate of 73% [30]. The addition of the ADC to standard 7 + 3 induction chemotherapy is tested within a large phase Ib (NCT02326584) study. Preliminary results have been reported for the first 42 patients of this study. The combination therapy resulted in grade 4 myelosuppression in all patients, but no increase in non-hematological AEs was reported compared to chemotherapy alone. Synergistic effects of HMAs and CD33-directed immunotherapy are supported by a high CR/CRi rate of 78% [31]. This could be due to HMA-induced increase in CD33 expression as well as increased sensitivity to toxin-induced DNA damage [28]. Based on the encouraging response data, a phase III study of SGN-CD33A in combination with azacitidine or decitabine for older patients with newly diagnosed AML (CASCADE study) has recently been initiated (NCT02785900). However, potential hepatotoxicity, including veno-occlusive disease (VOD), is a major concern, particularly in the combination of SGN-CD33A with allogeneic HSCT before or after the treatment. Both phase I studies discussed above have therefore been put on hold by the FDA to explore the incidence of VOD, while the CASCADE trial continues enrollment [32].

SGN-CD123A is a similar ADC with the antibody directed at CD123 instead of CD33. CD123 is more restrictively expressed in the healthy hematopoietic compartment, which might decrease on-target off-leukemia toxicities [24, 33]. This is being tested in the recently initiated phase I trial, which is planned to recruit 102 patients with r/r AML (NCT02848248).

ImmunoGen developed IMGN779, a CD33-directed monoclonal antibody conjugated to the novel DNA-alkylating molecule DGN462. Preclinical data demonstrated highly specific in vitro and in vivo cytotoxicity against primary AML cells, especially in samples with an *FLT-ITD* mutation [34, 35]. The combinatorial approach of IMGN779 with the PARP inhibitor Olaparib resulted in enhanced ex vivo activity and a decreased tumor burden in a xenograft mouse model [36]. A clinical phase I study in r/r AML is currently recruiting patients (124 patients planned, NCT02674763). Results of this study will show if there is any benefit over the usage of SGN-CD33A in terms of the risk-benefit ratio. Apart from the conjugation to toxins, monoclonal anti-CD33 antibodies have also been conjugated to radioisotopes. However, first clinical studies have demonstrated less promising results and most of these strategies are currently not further pursued [37, 38].

Taken together, the field of ADCs finally seems to recover from the huge setback it originally suffered after the voluntary withdrawal of GO in 2010. A lot of effort has been put into the optimization of the ADC technology, and clinical results from early trials demonstrate promising response rates. Results of randomized phase III trials are eagerly awaited in order to estimate the risk-benefit ratio between a potential increase in response rates and the discussed side effects due to on-target off-leukemia toxicities and toxin-induced hepatic toxicity. In order to increase target cell specificity of the therapy, alternative target antigens are being evaluated in preclinical (i.e., CLL-1, SAIL) [39–41] and early clinical studies (i.e., CD25, FLT3) [42, 43].

## T cell-recruiting antibody constructs for immunotherapy of AML

T cell-recruiting antibody constructs are a novel class of molecules composed of the single-chain variable fragments (scFv) of two antibodies of different specificity connected by a short peptide linker (Fig. 1c). Through simultaneous binding of a tumor-associated antigen and CD3ε in the T cell receptor complex, these small adapter molecules bring malignant cells and T cells in close proximity. The binding of CD3ε leads to T cell activation and expansion resulting in Granzyme B/perforin-mediated target cell lysis. The special feature of this strategy is that virtually any memory T cell can be recruited for target cell lysis irrespective of its specificity [44, 45]. Clinical proof of concept has been provided with blinatumomab (BLINCYTO®, AMGEN), a CD19/CD3 T cell-recruiting antibody construct. It was approved as the first in its class by the FDA in 2014 for r/r Ph-negative B-precursor ALL, after a clinical phase II trial demonstrated a CR/CRi rate of 43% after one or two cycles of therapy [9]. Very recently, the superiority of blinatumomab to conventional chemotherapy for patients with r/r B-precusor ALL was proven in a randomized phase III trial [46].

In AML, several T cell-recruiting antibody constructs are under preclinical and early clinical development (Table 2). Similar to the ADCs, the optimal antigen to target is still an open question. The sister molecule of blinatumomab, AMG 330, is a bispecific T cell engager (BiTE) construct targeting CD33 [25, 47]. The high inter- as well as intra-patient variations in CD33 expression levels might influence the success of targeted immunotherapy. Significantly lower expression has been demonstrated for CD34^+^/CD38^−^ leukemia-initiating cells (LICs) vs. AML bulk cells, but expression was still significantly higher compared to their healthy counterparts (CD34^+^/CD38^−^ normal hematopoietic stem cells). In preclinical studies, the preincubation of AML cells with AMG 330 and T cells prevented the subsequent engraftment of AML in NOD/SCID gamma null (NSG) mice. This suggests that the CD33 expression level of LICs is sufficient for elimination with T cell-recruiting constructs. Besides, it has been demonstrated in vitro that the CD33 expression level mainly influences kinetics of cytotoxicity, but not necessarily the response rate [25, 48]. Recently, an international, multicenter phase I trial for r/r AML patients (*n* = 50) was initiated (NCT02520427), but data are not yet available. Several other CD33-targeting antibody constructs that differ from AMG 330 in their molecular structure are currently evaluated in preclinical settings [12, 49, 50].

**Table 2:** Current clinical trials using T cell-recruiting antibody constructs for immunotherapy of AML

Study identifier	Study name	Antigen/target	Drug name	Combination therapy	Clinical phase	Indication (AML only)	Primary endpoints	(Estimated) Enrollment	Sponsor	Country	Study start	(Estimated) Completion date	Status
NCT02152956	Phase 1, first in human, dose escalation study of MGD006, a CD123 × CD3 dual affinity re-targeting (DART®) bi-specific antibody-based molecule, in patients with relapsed or refractory AML or intermediate-2/high risk MDS	CD123	MGD006	No	I	r/r AML	DLT	124	Macrogenics	USA, France, Germany, Italy, Netherlands	2014	2018	Recruiting
NCT02520427	A phase 1 first-in-human study evaluating the safety, tolerability, pharmacokinetics, pharmacodynamics and efficacy of AMG 330 administered as continuous intravenous infusion in subjects with relapsed/refractory acute myeloid leukemia	CD33	AMG 330	No	I	r/r AML	DLT, toxicity	50	AMGEN	USA, Germany, Netherlands	2015	2018	Recruiting
NCT02715011	A phase 1, first-in-human, open-label, dose escalation study of JNJ-63709178, a humanized CD123 × CD3 DuoBody in subjects with relapsed or refractory AML	CD123	JNJ-63709178	No	I	r/r AML	DLT, toxicity	60	Janssen Research & Development	USA, Australia, Belgium, Germany	2016	Unknown	Suspended
NCT02730312	A phase 1 multiple dose study to evaluate the safety and tolerability of XmAb®14045 in patients with CD123-expressing hematologic malignancies	CD123	Xmab14045	No	I	Primary or secondary AML	MTD, toxicity	66	Xencor	USA	2016	2018	Recruiting
NCT03038230	A phase 1, multinational study of MCLA-117 in acute myelogenous leukemia	CLL-1	MCLA-117	No	I	r/r AML, newly diagnosed elderly untreated AML patients	DLT, toxicity	50	Merus N.V.	Belgium, France, Italy, Netherlands	2016	2018	Recruiting

To reduce on-target off-leukemia toxicity, alternative AML-associated targets are being explored. CD123 has a lower level of expression on healthy hematopoietic cells compared to CD33 [24, 33]. Therefore, several T cell-recruiting antibody constructs targeting CD123 have been developed and are currently in early clinical studies. One of these constructs is MGD006, developed by MacroGenics. In contrast to the BiTE technology, dual-affinity re-targeting (DART) molecules are composed of heavy and light chain variable domains of two antigen-binding specificities (A + B) on two independent polypeptide chains (VL_A_-VH_B_-VL_B_-VH_A_), which are stabilized through an additional C-terminal bridge [51, 52]. Encouraging preclinical data in terms of cytotoxicity against primary AML cells [53] and safe and well-tolerated infusion of MGD006 in cynomolgus monkeys [54] paved the way for the clinical development in a multicenter phase I study of 124 relapsed/refractory AML patients (NCT02152956).

XmAb14045, developed by Xencor, is a structurally distinct anti-CD123 T cell-recruiting antibody construct in early clinical development. The XmAb technology ensures structural stability and an extended serum half-life through the retention of an inactive Fc part. Preclinical studies in cynomolgus monkeys showed rapid clearance of CD123^+^ cells from the bone marrow as well as from the periphery [55]. These studies formed the basis for the initiation of a clinical phase I study for the evaluation of safety and tolerability of Xmab14045 in 66 patients with CD123-expressing hematological malignancies including primary and secondary AML (NCT02730312).

JNJ-63709178, a CD123/CD3 humanized IgG4 antibody has been developed by Genmab using their DuoBody technology. Preclinical studies in vitro and in vivo showed highly specific T cell activation and targeting of primary AML cells [56, 57], which lead to the initiation of a phase I study in relapsed/refractory AML (*n* = 60, NCT02715011). Currently, the study is on hold because of the occurrence of undisclosed adverse events.

CLL-1 is a novel target antigen in AML characterized by its high expression on AML bulk cells as well as LICs [58, 59]. Recently, a bispecific CLL-1/CD3 antibody construct (MCLA-117) has been developed by Merus B.V. MCLA-117 induced target antigen-specific cytotoxicity against primary AML cells at low E:T ratios using either allogeneic or autologous T cells. This led to the initiation of a clinical phase I trial in r/r or elderly, previously untreated AML patients (NCT03038230, *n* = 50) [60].

Results of the ongoing trials are awaited to see if the success in ALL will translate into the setting of AML. A potential future strategy could be to use the evolving antibody technology to simultaneously target two different AML-associated antigens in order to increase specificity [61]. Apart from that, lots of effort has been put into optimization of the antibody technology to increase safety. The Probody™ technology by CytomX uses antigen-binding site masking peptides attached to antibody constructs by substrate-cleavable linkers. In the tumor microenvironment, linkers are cleaved by highly active proteases generating effective immunotherapeutic agents directly at the tumor site [62]. Recently, an EGFR/CD3 Probody™ has shown promising results in terms of efficacy and increase in therapeutic window in preclinical studies in vitro and in vivo. As the technology relies on tumor site-specific protease activity, it remains to be determined if this approach is also feasible in acute leukemia [63].

Independently of considerations about the optimal target antigen, we are only at the beginning of understanding the exact mechanism of action of those antibody constructs and resistance mechanisms that potentially evolve upon T cell activation. Despite the promising response rate of 43% using blinatumomab in heavily pre-treated ALL patients, reasons for resistance in the remaining patients have not been resolved. Only few biomarkers for response have been determined so far, e.g., in case of the blinatumomab studies, the percentage of blasts in the bone marrow and the degree of T cell expansion [9, 64]. PD-L1 upregulation on AML cells upon T cell activation has been suggested as a potential resistance mechanism in an ex vivo system [48] and in a case report of a blinatumomab-refractory B-precursor ALL patient [65]. Addition of a checkpoint inhibitor to T cell-recruiting antibodies might help to circumvent resistance. A clinical study testing this concept by addition of an anti-PD1 antibody with or without an anti-CTLA4 antibody to blinatumomab for the treatment of r/r ALL patients has been initiated, but is not yet open for patient recruitment (NCT02879695).

## CAR T cells for immunotherapy of AML

Circumventing T cell exhaustion, anergy and senescence, CAR T cells take the technology of T cell-recruiting antibody constructs one step further and have already shown promising clinical results in various hematologic malignancies. CARs are genetically engineered cell membrane-bound receptors that combine extracellular antibody binding and intracellular effector cell signaling, thereby enabling both MHC-independent antigen binding and highly potent cytotoxic effector cell function (Fig. 1d). Since the first generation of CARs in 1989 [66], the introduction of costimulatory domains (mainly CD28 or 4-1BB) in so-called second-generation CAR constructs greatly improved their anti-tumor effector function and paved their way into clinical trials [67].

To date, the most prominent target antigen for CAR T cell therapy is CD19, due to its restrictive expression pattern and good safety profile. Groundbreaking early clinical trial results could be achieved for various B cell malignancies. In r/r B-ALL, treatment with anti-CD19 4-1BB-costimulatory CAR T cells achieved MRD-negative CR rates of 86% for 29 patients [68]. These are outstanding clinical results, considering the heavily pretreated patient population that was included: in the median, patients had received three prior intensive chemotherapy regimens, and more than one third had relapsed after prior allogeneic HSCT. In another recently published trial, treatment with anti-CD19 CD28-costimulatory CAR T cells showed great clinical efficacy with CR rates of 57% in seven patients with DLBCL refractory to at least three prior lines of therapy [69]. As of November 1, 2016, 1135 patients have been treated with anti-CD19 genetically engineered TCR/CAR T cells [70], leading to high expectations for patients with no therapeutic options until now. Accordingly, there are currently 87 open clinical phase I or II trials involving anti-CD19 CAR T cells in B cell malignancies (ClinicalTrials.gov, last update 03/07/2017).

Despite these promising early results and the rapidly expanding number of anti-CD19 CAR T cell trials, this novel drug format is still incompletely understood and cannot generally be considered safe. In March 2017, Juno announced to shut down development of anti-CD19 CD28-costimulatory JCAR015 CAR T cells and to close their phase II ROCKET trial in r/r adult ALL, after five treatment-related deaths had occurred due to CAR T cell-mediated neurotoxicity [71]. As “living drugs,” the in vivo effect of CAR T cells may be dependent on different conditioning chemotherapy regimens, CAR T cell manufacturing protocols and costimulatory domains. Unfortunate combinations of these variables may promote rapid in vivo expansion of CAR T cells with the potential to induce severe systemic and neurological side effects.

Translating CAR T cell therapy to AML is complicated again by the non-restricted expression of AML-associated antigens. Given that current CAR T cell constructs can persist beyond 4 years in the human body [72], several strategies are being explored to circumvent unwanted on-target off-leukemia toxicity, particularly long-term myeloid cell aplasia. Similar to ADCs and T cell-recruiting antibody constructs, the identification of AML-specific target antigens or antigen combinations would be one way to improve safety of future CAR T cell approaches in AML. To date, several target antigens for AML CAR T cell therapy are under preclinical and clinical investigation.

CD33 is the most prominent target antigen for CAR T cells in preclinical trials due to its high and persistent expression in the majority of AML patients [24, 73]. In an in vivo model of AML-xenotransplanted NSG mice, treatment with anti-CD33 CAR T cells resulted in marked reduction of leukemic burden and prolonged survival [74]. However, significant on-target off-leukemia toxicity with reduction of myeloid lineage and hematopoietic stem cells was observed. In another in vivo model of AML-xenotransplantated NSG mice, treatment with only transient CAR expression via electroporation of T cells with anti-CD33 CAR-encoding RNA resulted in similar, but only transient cytotoxicity [75]. Application of CAR T cells directed against CD123 as an alternative target in an in vivo model with AML-xenotransplanted mice resulted in significant reduction of leukemic burden and prolonged survival with only limited on-target off-leukemia toxicity and unaffected healthy hematopoiesis [76–79]. In contrast, eradication of normal human myelopoiesis was demonstrated in another in vivo mouse study with anti-CD123 CAR T cells [80]. Interestingly, modifying the anti-CD123 scFv by utilizing V_H_ and V_L_ chains from different monoclonal antibodies could reduce myelotoxicity in an AML mouse model [79]. This conflicting data indicates that variations in antibody clone, costimulatory domain, effector cells, and model system might account for vastly different outcomes. Fine-tuning the development process of CAR T cells might be able to provide differential recognition of target antigens on leukemic vs. healthy cells.

Other potential target antigens identified in preclinical studies include CD44v6 [81], CLL1 [82], FLT3 [83], FRβ [84], LeY [85], NKG2D [86], and PR1/HLA-A2 [87].

To date, only one very small trial evaluating anti-LeY CAR T cells (CTX08-0002) in r/r AML has been completed. None of the four treated patients developed grade 3 or 4 toxicity, and infused CAR T cells persisted for up to 10 months. One patient with active leukemia responded with transient reduction in blast count before progression 1 month later. All patients relapsed 28 days to 23 months after adoptive CAR T cell transfer [88]. Currently, there are four open phase I clinical trials that evaluate the application of CAR constructs in r/r AML (Table 3). One trial recruiting in China is including patients with r/r AML for treatment with anti-CD33 CAR cytokine-induced killer (CIK) cells (NCT01864902). So far, there has only been a report of one patient within this trial who showed a transient decrease in blast count while suffering from cytokine release syndrome and pancytopenia [89]. Trial completion is estimated to be in 2017. Two other trials evaluate lentivirally transduced or mRNA-electroporated anti-CD123 CAR T cells, respectively (NCT02159495, NCT02623582), however, the latter one has been prematurely terminated. Until now, no results have been published. Another phase I trial utilizing allogeneic “off-the-shelf” anti-CD123 CAR T cells (UCART123) was recently opened (NCT03190278 [90]). And finally, a trial applying CAR T cells directed at NKG2D ligands to patients with r/r AML, MDS, and multiple myeloma is estimated to be completed in 2017, but results are still pending (NCT02203825).

**Table 3:** Current clinical trials using CAR T cells for immunotherapy of AML

Study identifier	Study name	Target	Designation	Generation	Costim. domain	Transduction method	Median dosage	Conditioning chemotherapy	Clinical phase	Indication	Primary endpoints	(Estimated) Enrollment	Sponsor	Country	Study start	(Estimated) Completion date	Status
NCT01864902	Treatment of Relapsed and/or Chemotherapy Refractory CD33 Positive Acute Myeloid Leukemia by CART-33 (CART33)	CD33	CART-33	2nd	4-1BB	Lentiviral	4.26 × 10^8^ CAR T cells	n.a.	I/II	r/r AML or AML in CR2 or later if not a candidate for allo-HSCT; CD33 expression	Toxicity	10 (1 patient reported)	Chinese PLA General Hospital	China	2013	2017	Recruiting
NCT02159495	Genetically Modified T-cell Immunotherapy in Treating Patients With Relapsed/Refractory Acute Myeloid Leukemia and Persistent/Recurrent Blastic Plasmacytoid Dendritic Cell Neoplasm	CD123	CD123R(EQ) 28Z/EGFRt	2nd	CD28	Lentiviral	Variable	Cyclophosphamide +/− fludarabine +/− etoposide	I	r/r AML	DLT, toxicity	30	City of Hope Medical Center	USA	2015	2017	Recruiting
NCT02203825	Safety Study of Chimeric Antigen Receptor Modified T-cells Targeting NKG2D-Ligands	NKG2D-ligands	CM-CS1 T-cells	2nd	DAP10	Retroviral	1 × 10^6^ − 3 × 10^9^ CAR T cells/kg	n.a.	I	r/r MDS-RAEB, r/r AML, r/r MM	Toxicity, feasibility	12	Celyad	USA	2015	2017	Active, not recruiting
NCT03190278	Study Evaluating Safety and Efficacy of UCART123 in Patients With Acute Myeloid Leukemia (AML123)	CD123	UCART123	n.a.	n.a.	n.a.	6.25 × 10^5^ − 6.25 × 10^6^ CAR T cells/kg	n.a.	I	r/r AML	Safety, efficacy	156	Cellectis S.A.	USA	2017	2021	Recruiting

Novel CAR designs are explored to increase the specificity and to improve safety profiles. In preclinical in vivo models, dual-targeting approaches targeting two independent leukemia-associated antigens were shown to provide increased specificity accompanied by reduced off-leukemia toxicity [91] and to prevent antigen escape mechanisms [92]. In vitro, it was demonstrated that dual targeting of CD33 and CD123 was superior to monospecific approaches in terms of specific cytotoxicity [93]. Further preclinical investigation and translation of dual-targeting strategies into clinics could contribute to efficacy and safety in CAR T cell therapy in AML where target specificity remains a major issue. On-target off-leukemia toxicity could also be further reduced by fine-tuning of CAR density and CAR binding affinity [94]. In light of safety concerns due to unrestricted in vivo CAR T cell expansion and activation, methods of selective CAR T cell depletion are currently being investigated. Integration of so-called suicide gene systems into CAR constructs could act as safety switches enabling rapid on-demand elimination of CAR T cells that would otherwise turn uncontrollable. These suicide gene systems can be based on enzymatic activation of cytotoxic prodrugs, antibody-based targeting of overexpressed surface antigens, or pharmacological induction of apoptosis via inducible caspase 9 which is already tested in clinical phase I CAR T cell trials (NCT03016377 [95]).

## Checkpoint inhibitors for immunotherapy of AML

In contrast to the immunotherapeutic concepts discussed so far, monoclonal antibodies against checkpoint molecules are applied with the idea to unleash pre-existing anti-tumor T cell responses (Fig. 1e). Within recent years, checkpoint inhibition has probably become the single biggest hype in cancer immunotherapy, primarily in solid oncology, but meanwhile, also finding its way into hematology [96]. Most prominently within hematologic diseases, anti-PD-1 antibodies show remarkable success in Hodgkin’s lymphoma and are tested in various non-Hodgkin lymphomas. However, there is growing evidence from in vitro experiments and murine models that this strategy could also be applied to AML [96].

Only one clinical study applying a checkpoint antibody as a monotherapy to AML patients has been published so far. Eighteen patients with various hematologic malignancies, including eight patients with AML, were treated with the anti-PD-1 antibody pidilizumab within a phase I study. The antibody was shown to be safe and well tolerable, and one of the AML patients showed a minimal response manifested by a decrease in peripheral blasts from 50 to 5% [97]. A phase I study testing the CTLA-4 antibody ipilimumab in various malignancies including 12 patients with AML has long been completed, but to our knowledge, specific results for AML patients have not been published (NCT00039091, Table 4). Another phase I study, in which ipilimumab was applied to 54 patients with refractory AML, MDS, or chronic myelomonocytic leukemia (CMML), has finished recruiting, but results have not yet been reported (NCT01757639). And three phase II studies (NCT02275533, NCT02532231, NCT02708641) are studying the effect of PD-1 inhibition with either nivolumab or pembrolizumab as a monotherapy on prevention of relapse in remission.

**Table 4:** Current clinical trials using checkpoint inhibitors for immunotherapy of AML

Study identifier	Study name	Antigen/target	Drug name	Combination therapy	Clinical phase	Indication (AML only)	Primary endpoints	(Estimated) Enrollment	Sponsor	Country	Study start	(Estimated) Completion date	Status
NCT00039091	Monoclonal antibody therapy in treating patients with ovarian epithelial cancer, melanoma, acute myeloid leukemia, myelodysplastic syndrome, or non-small cell lung cancer	CTLA-4	Ipilimumab	n.a.	I	AML with different recurrent mutations or recurrent AML	Toxicity	12 (AML only)	National Cancer Institute (NCI)	USA	2002	2007	Terminated
NCT01757639	Ipilimumab in treating patients with relapsed or refractory high-risk myelodysplastic syndrome or acute myeloid leukemia	CTLA-4	Ipilimumab	n.a.	I	Refractory AML	Toxicity, regulatory T cells	54 (AML + MDS + CMML)	National Cancer Institute (NCI)	USA	2012	2016	Active, not recruiting
NCT02275533	Nivolumab in eliminating minimal residual disease and preventing relapse in patients with acute myeloid leukemia in remission after chemotherapy	PD-1	nivolumab	n.a.	II	AML in first remission; no eligibility for allo-HSCT	Clinical response (RFS)	80	National Cancer Institute (NCI)	USA	2015	2019	Recruiting
NCT02397720	Study of Nivolumab (BMS-936558) in Combination With 5-azacytidine (Vidaza) for the Treatment of Patients With Refractory/ Relapsed Acute Myeloid Leukemia and Newly Diagnosed Older Acute Myeloid Leukemia (AML) (>65 Years) Patients	PD-1	Nivolumab	Azacitidine	II	r/r AML or newly diagnosed older AML patients	MTD	110	M.D. Anderson Cancer Center	USA	2015	2018	Recruiting
NCT02464657	Study of Idarubicin, Cytarabine, and Nivolumab in Patients With High-Risk Myelodysplastic Syndrome (MDS) and Acute Myeloid Leukemia (AML)	PD-1	Nivolumab	Idarubicin, cytarabine	I/II	De novo AML	MTD	75	M.D. Anderson Cancer Center	USA	2015	2018	Recruiting
NCT02532231	Nivolumab in Acute Myeloid Leukemia (AML) in Remission at High Risk for Relapse	PD-1	Nivolumab	n.a.	II	AML in remission with high risk of relapse	Clinical response (RFS)	30	M.D. Anderson Cancer Center	USA	2015	2018	Recruiting
NCT02708641	A phase II study of pembrolizumab as post-remission treatment of patients ≥60 with AML	PD-1	Pembrolizumab	n.a.	II	AML patients ≥60 years in CR; no eligibility for allo-HSCT	Toxicity, clinical response (time to relapse)	40	Alison Sehgal, MD, MS	USA	2016	2021	Not yet recruiting
NCT02768792	High-dose cytarabine followed by pembrolizumab in relapsed/refractory AML	PD-1	Pembrolizumab	High-dose cytarabine	II	r/r AML	Clinical response (CR rate)	37	UNC Lineberger Comprehensive Cancer Center	USA	2016	2021	Recruiting
NCT02771197	Lymphodepletion and anti-PD-1 blockade to reduce relapse in AML patient not eligible for	PD-1	Pembrolizumab	Fludarabine, melphalane, auto-SCT	II	Non-favorable risk AML in CR	Clinical response (2-y-RR)	20	Northside Hospital, Inc.	USA	2016	2020	Recruiting
NCT02775903	An efficacy and safety study of azacitidine subcutaneous in combination with durvalumab (MEDI4736) in previously untreated subjects with higher-risk myelodysplastic syndromes (MDS) or in elderly subjects with acute myeloid leukemia (AML)	PD-L1	Durvalumab	Azacitidine	II	De novo AML or sAML or tAML in elderly patients	Clinical response (RR)	110 (AML alone)	Celgene Corporation	USA, Canada and various European countries	2016	2019	Recruiting
NCT02845297	Phase 2 study of azacitidine in combination with pembrolizumab in relapsed/refractory acute myeloid leukemia (AML) patients and in newly diagnosed older (≥65 years) AML patients	PD-1	Pembrolizumab	Azacitidine	II	r/r AML	MTD	40	Sidney Kimmel Comprehensive Cancer Center	USA	2016	2020	Recruiting
NCT02890329	Ipilimumab and decitabine in treating patients with relapsed or refractory myelodysplastic syndrome or acute myeloid	CTLA-4	Ipilimumab	Decitabine	I	r/r AML	MTD	48	National Cancer Institute (NCI)	USA	2017	2019	Not yet recruiting
NCT02890329	Ipilimumab and decitabine in treating patients with relapsed or refractory myelodysplastic syndrome or acute myeloid	CTLA-4	Ipilimumab	Decitabine	I	r/r AML or de novo AML in elderly patients	Toxicity, MTD	48 (AML + MDS)	National Cancer Institute (NCI)	USA	2017	2019	Not yet recruiting
NCT02892318	A study evaluating the safety and pharmacology of atezolizumab administered in combination with immunomodulatory agents in participants with acute myeloid leukemia (AML)	PD-L1	Atezolizumab	Guadecitabine, possibly other immunomodulatory agents	I	r/r AML or de novo AML in elderly patients	Toxicity, clinical response (CR, CRi, CRp, duration of response)	40	Hoffmann-La Roche	USA	2016	2019	Recruiting
NCT02953561	Avelumab (antiPDL1) and azacytidine in acute myeloid leukemia (AML)	PD-L1	Avelumab	Azacitidine	I/II	r/r AML	Toxicity	52	M.D. Anderson Cancer Center	USA	2017	2020	Not yet recruiting
NCT02996474	Pembrolizumab and decitabine for refractory or relapsed acute myeloid leukemia	PD-1	Pembrolizumab	Decitabine	I/II	r/r AML	Feasibility	15	National Heart, Lung, and Blood Institute (NHLBI)	USA	2016	2019	Not yet recruiting

While the results of these studies have to be awaited to judge the potential of checkpoint inhibitors as a monotherapy for AML, various combination therapies are already tested in clinical trials. A phase II study is combining lymphodepletion with a fludarabine/melphalane regimen followed by autologous stem cell transplantation with anti-PD-1 therapy with the goal to reduce relapse rates in non-favorable AML patients in remission (NCT02771197). The combination of standard high-dose cytarabine with anti-PD-1 therapy is tested as a salvage therapy in a phase II study planned to recruit 37 patients with r/r AML (NCT02768792). And a phase I/II study analyzes the maximal tolerable dose of an anti-PD-1 antibody in addition to idarubicin and cytarabine for induction of de novo AML (NCT02464657). No results for any of these studies have been reported so far. The combination of a PD-1 antibody with a vaccination strategy based on AML DC hybridoma is described in the DC chapter below (NCT01096602, Table 5).

A high interest is currently generated by the idea to combine checkpoint inhibition with HMAs. The evaluation of PD-1 as well as PD-L1 expression in patients with MDS or AML receiving HMAs showed upregulation of both markers on mRNA level [98]. Therefore, several trials are evaluating the efficacy of HMAs combined with either CTLA-4, PD-1, or PD-L1 blocking antibodies (Table 4). First results for this strategy within a phase Ib/II study combining the PD-1 blocking antibody nivolumab with azacitidine in patients with r/r AML have recently been presented. Toxicity was comparable with other trials using checkpoint blockade, and outcomes have been encouraging with a median overall survival of 9.3 months in this study with a predominantly poor-risk patient population [99].

Taken together, checkpoint inhibition in AML is still in its infancy, and results of the currently ongoing trials have to be awaited before further conclusions about the applicability of this concept to AML and the existence of any AML-specific side effects of checkpoint inhibition can be drawn. Combination therapies including checkpoint inhibitors, particularly with HMAs, might turn out to be an important step forward.

## Dendritic cell vaccination for immunotherapy of AML

Vaccination strategies have the purpose to prime new or enhance pre-existing antigen-specific immune responses. DCs are highly eligible for the induction of tailored, strong, and durable responses (Fig. 1f). This is of particular importance for the treatment of tumor entities with low endogenous immune responses, such as AML. In spite of the high costs and efforts accruing for the production of this patient-specific cellular therapy, DC-based vaccination strategies for the treatment of AML are therefore actively pursued. Important variables in these studies are source of DC precursors, DC maturation protocol, target antigen, way of antigen loading route of application, and interval of application [100]. While monocyte-derived DCs are used in the majority of studies and are considered to induce the strongest immune responses, alternative DC-like constructs are also applied [1].

Recently, an interesting clinical trial has been published presenting 17 AML patients that were vaccinated in CR with a hybridoma of AML cells and autologous DCs [101]. The vaccination was well tolerated, and a considerable increase in leukemia-specific T cells was found that persisted for more than 6 months. High relapse-free survival was described, but a strong selection bias for long-term survivors currently impedes further interpretations. This patient cohort is part of a larger study that is designated to analyze the combinatorial effect of PD-1 blockade with the described vaccination strategy (NCT01096602, see Table 5). However, data for the combination therapy has not been released.

**Table 5:** Current clinical trials using dendritic cell vaccination for immunotherapy of AML

Study identifier	Study name	Type of vaccine	Antigen/target	Antigen source	Combination therapy	Clinical phase	Indication (AML only)	Primary endpoints	(Estimated) Enrollment	Sponsor	Country	Study start	(Estimated) Completion date	Status
NCT00100971	Vaccine therapy in treating patients with acute myeloid leukemia	Fusion of dendritic and leukemic cells	Multiple	Inherent	n.a.	I	De novo AML	MTD, toxicity	9	Boston Medical Center	USA	2004	2007	Terminated early due to slow accrual
NCT00136422	Study of vaccination with autologous acute myeloblastic leukemia cells in patients with advanced myelodysplasia or acute myelogenous leukemia	Lethally irradiated and genetically modified autologous AML cells	Multiple	Inherent	n.a.	I	r/r AML or de novo AML in non-fit patients	Feasibility	30	Dana-Farber Cancer Institute	USA	2000	2006	Completed
NCT00510133	A study of active immunotherapy with GRNVAC1 in patients with acute myelogenous leukemia (AML)	Monocyte-derived dendritic cells	hTERT	mRNA	n.a.	II	AML in CR1 or CR2	Feasibility	21	Asterias Biotherapeutics, Inc.	USA	2007	2014	Completed
NCT00514189	Feasibility study of acute myelogenous leukemia mRNA plus lysate-loaded dendritic cell vaccines	Monocyte-derived dendritic cells	Multiple	AML mRNA + lysate	n.a.	I	De novo AML with non-favorable cytogenetics or AML in first relapse	Feasibility, toxicity, immunogenicity	2	M.D. Anderson Cancer Center	USA	2007	2009	Terminated early due to slow accrual
NCT00834002	Dendritic cell vaccination for patients with acute myeloid leukemia in remission (CCRG 05–001)	Monocyte-derived dendritic cells	WT1	mRNA	n.a.	I/II	AML in CR/PR with WT1 overexpression and high risk of relapse	Feasibility, toxicity	10	University Hospital, Antwerp	Belgium	2005	2008	Completed
NCT00963521	Vaccine therapy in treating patients with acute ,myeloid leukemia in complete	In vitro-differentiated leukemic blasts	Multiple	Inherent	n.a.	I	AML in CR (CR2 or later)	Toxicity	10	Institut Paoli-Calmettes	France	2008	2011	Completed
NCT00965224	Efficacy of dendritic cell therapy for myeloid leukemia and myeloma	Monocyte-derived dendritic cells	WT1	mRNA	n.a.	II	AML in CR with high risk of relapse	Immunogenicity, molecular response	50	University Hospital, Antwerp	Belgium	2010	2014	Enrolling by invitation
NCT01096602	Blockade of PD-1 in conjunction with the dendritic cell/AML vaccine following chemotherapy induced	Dendritic cell AML fusion vaccine	Multiple	Inherent	PD1 blockade, GM-CSF	II	AML at initial diagnosis or at first relapse	Toxicity	63	Beth Israel Deaconess Medical Center	USA	2010	2017	Active, not recruiting
NCT01146262	Vaccination by leukemic apoptotic corpse autologous pulsed dendritic cells for acute myelogenous leukemia (AML) patients in first or second complete remission (CR) (CDlaM)	Monocyte-derived dendritic cells	Multiple	AML apoptotic corpse	n.a.	I/II	AML in CR2 or refractory AML or de novo AML with unfavorable cytogenetics; no eligibility for allo-HSCT	Toxicity	5	Nantes University Hospital	France	2009	2017	Active, not recruiting
NCT01373515	Leukemic dendritic cell vaccination in patients with acute myeloid leukemia	Dendritic-like cells generated from standardized allogeneic AML cells	Multiple	Inherent	n.a.	I/II	AML in CR2 or relapsed AML or de novo AML; no eligibility for intensive therapy	Feasibility, toxicity	12	DCPrime BV	Netherlands	2011	2013	Completed
NCT01686334	Efficacy study of dendritic cell vaccination in patients with acute myeloid leukemia in remission (WIDEA)	Monocyte-derived dendritic cells	WT1	mRNA	n.a.	II	AML in CR or Cri; WT1 overexpression	Clinical response (RR, DFS, OS)	138	University Hospital, Antwerp	Belgium	2012	2020	Recruiting
NCT01734304	DC vaccination for postremission therapy in AML	Monocyte-derived dendritic cells	WT1, PRAME	mRNA	n.a.	I/II	AML in CR or CRi with non-favorable risk profile; no eligibility for allo-	Feasibility, toxicity	20	Ludwig-Maximilians-University of Munich	Germany	2012	2017	Recruiting
NCT02405338	DC vaccination for postremission therapy in AML	Monocyte-derived dendritic cells	WT1, PRAME	mRNA	n.a.	I/II	AML in CR or Cri; WT1 overexpression; no eligibilty for allo-HSCT	Feasibility, toxicity	20	Medigene AG	Norway	2015	2019	Recruiting

DCPrime uses an off-the-shelf product based on a precursor human dendritic cell line. This platform was tested in a phase I/II study for AML patients (NCT01373515), and vaccinations were well tolerated with induction of multi-functional immune responses, resulting in the preparation of a multi-center phase II study. However, there is no full publication of the study results available at present. To our knowledge, no other clinical trial is currently recruiting patients for vaccination concepts with DC-like cells, as a study based on a fusion concept has been terminated early due to slow accrual (NCT00100971), and two studies using modified leukemic blasts (NCT00136422, NCT00963521) have been completed, but their results have not been published (see Table 5).

Monocyte-derived DCs loaded with various antigens are the most commonly used source for DC vaccination trials. Five clinical studies are currently active or recruiting. A small French study (*n* = 5) uses AML apoptotic corpses to load DCs (NCT01146262). A group in Belgium that has already completed a phase I/II study on vaccination with *WT1* mRNA-loaded DCs for 10 AML patients in remission with high risk of relapse demonstrating immunological as well as clinical responses [102] is now conducting a phase II study testing the induction of immune and molecular responses by vaccination with *WT1* mRNA-loaded DCs for AML as well as chronic myeloid leukemia and multiple myeloma patients (NCT 00965224). Besides, the same group also conducts a large (estimated enrollment, 138 patients) randomized phase II study on AML patients in CR/CRi with WT1 overexpression with the goal to determine clinical effects of DC vaccination in terms of relapse rate, disease-free survival, and overall survival (NCT01686334). Results of this study are eagerly awaited, but are not to be expected before 2020.

Our group in Munich has developed a protocol for the generation of DCs by the use of a TLR7/8 agonist [103, 104]. These DCs show improved immunogenicity compared to conventional monocyte-derived DCs [105]. We are currently conducting a phase I/II proof-of-concept study using this type of DCs loaded with mRNA encoding *WT1* and *PRAME* for intradermal vaccination of AML patients in CR with a non-favorable risk profile (NCT01734304) [106]. Preliminary results for 13 patients have already been reported at ASH, showing that DC generation is feasible, that their application is safe with delayed-type hypersensitivity reactions at the injection sites, but no serious adverse events, and that novel immune responses to both antigens can be induced. Immune responses were markedly increased by combination of DC vaccination with azacitidine within an individual treatment attempt [107]. A very similar study is conducted by our collaborators in Norway (NCT02405338).

Besides current clinical studies, a few interesting new developments in the field of DCs in the context of AML immunotherapy have been described in the past 2 years. In an effort to further optimize the immunostimulatory capacities of monocyte-derived DCs, electroporation of mRNA encoding both for IL-15 and for IL-15 receptor alpha was shown to result in enhanced NK cell activation [108]. Besides, evidence was provided that monocyte-derived DCs express RHAMM independent of RNA electroporation at a level high enough to induce RHAMM-specific T cells [109].

In conclusion, current data suggests that DC vaccination is particularly successful at inducing novel immune responses. Combining this approach with checkpoint inhibition or immunomodulating agents including HMAs in order to further enhance the immune responses seems an interesting way to follow.

## Conclusions

Immunotherapy of cancer has made unprecedented progress in the past few years. While novel immunotherapeutic strategies have already moved into standard clinical practice for various solid cancers as well as selected hematological neoplasms including ALL, a similar development is lagging behind for the treatment of AML. However, different immunotherapeutic concepts are currently being evaluated in clinical trials, with some promising results already published and a lot more of interesting studies expected to be completed within the next couple of years.

The lack of an appropriate target antigen with a restricted expression pattern similar to CD19 or CD20 for B cell neoplasms is a major obstacle for the application of targeted immunotherapy in AML. This problem is shared by ADCs, T cell-recruiting antibody constructs and CAR T cell constructs, where promising leukemia-specific responses seen in early clinical trials are often accompanied by severe on-target off-leukemia toxicity to the myeloid compartment. CD33 and CD123 are the major target antigens of constructs in clinical development so far. Results of the ongoing clinical trials need to be awaited in order to weigh potential benefits vs. side effects. In order to prospectively reduce on-target off-leukemia toxicities, several strategies are followed: The identification of novel leukemia-associated antigens could provide more specific targets. Comprehensive transcriptomic and proteomic analysis is ongoing to fully characterize the AML surfaceome [110]. Alternatively, leukemia-specific neoantigens arising from AML-associated mutations should be further evaluated as source of novel target molecules. Furthermore, dual-targeting approaches could improve treatment specificity while relying on combinations of already known AML-associated antigens.

ADCs have already proven their therapeutic potential in AML. Results of currently running clinical trials will help to identify the optimal clinical setting and to better estimate the risk-benefit ratio. In contrast, T cell-recruiting antibodies and CAR T cell constructs are still in the early phase of clinical development for the therapy of AML, with several currently running phase I trials studying the feasibility and toxicity of their application. Activation of endogenous T cell responses through checkpoint blockade and/or DC vaccines appears to be safe, but has yet to demonstrate its clinical potency when used as a monotherapy for the treatment of AML. Different combinations including HMAs to modulate immune responsiveness appear suitable and are increasingly being tested.

While immunotherapy in AML is complicated by different characteristics including lack of an AML-specific target antigen, low mutational burden resulting in low endogenous immune responses and intrinsic resistance mechanisms of the leukemic blasts against immune responses, remarkable progress has been made with different strategies in the past few years. Hope is high that alternative immunotherapeutic strategies with less treatment-related morbidity and mortality compared to allogeneic HSCT will move into clinical practice within the coming years. Still, many further steps have to be taken before the vision of an individualized immunotherapy for each AML patient based on risk factors and biomarkers can become clinical reality.

## Abbreviations

ADC: Antibody-drug conjugate
AE: Adverse event
ALL: Acute lymphoblastic leukemia
AML: Acute myeloid leukemia
BiTE: Bispecific T cell engager
CAR: Chimeric antigen receptor
CIK: Cytokine-induced killer
CMML: Chronic myelomonocytic leukemia
CR: Complete response
CRi: Complete remission with incomplete recovery
DART: Dual-affinity re-targeting
DC: Dendritic cell
FDA: Food and Drug Administration
GO: Gemtuzumab ozogamicin
HMA: Hypomethylating agent
HSCT: Hematopoietic stem cell transplantation
LIC: Leukemia-initiating cell
MDS: Myelodysplastic syndrome
NSG: NOD/SCID gamma null
ORR: Overall response rate
OS: Overall survival
r/r: Relapsed or refractory
RCT: Randomized controlled trial
scFv: Single-chain variable fragment
VOD: Veno-occlusive disease
